# Let’s Talk About Single Men: A Qualitative Investigation of Never Married Men’s Experiences of Singlehood

**DOI:** 10.1007/s11199-023-01380-y

**Published:** 2023-05-18

**Authors:** Marta Mrozowicz-Wrońska, Kamil Janowicz, Emilia Soroko, Katarzyna Adamczyk

**Affiliations:** 1 Faculty of Psychology and Cognitive Science, Adam Mickiewicz University, ul. A. Szamarzewskiego 89/AB, Poznań, 60-568 Poland; 2 Center for Research on Personality Development, SWPS University, Poznań, Poland

**Keywords:** Never-married, Single men, Singlehood, traditional masculinity, Masculine norms, Gender stereotypes, Thematic qualitative analysis

## Abstract

Existing research on singlehood has largely focused on the experiences of single women, and little is known about singlehood among men. The current investigation examined the experience of long-term singlehood through individual, semi-structured interviews with 22 never-married single men living in Poland who were aged 22–43 years. Thematic analysis revealed five key themes: (1) the sense of being deficient—is there something wrong with me?; (2) navigating outside the dominant discourse of traditional masculinity, marriage and family; (3) the benefits and downsides of singlehood; (4) adaptation to singlehood; and (5) the dilemma between waiting and actively searching for a romantic partner. An analysis of single men’s narratives revealed that men experience their single status in the context of their various needs and hopes and as a status that determines their adult life course. This study contributes to the singlehood literature, highlighting the complexity of singlehood for men and the importance of traditional masculinity norms in experiencing long-term singlehood.

These findings challenge stereotypical and unrealistic views of singlehood among men and have practical implications for psychotherapists, counsellors and educators working with single men.

In recent decades, the role of marriage in people’s lives has been diminishing, and the number of single individuals has been rising (Bühler & Nikitin, 2020; Girme et al., 2022). Despite the acknowledgment of the importance of marriage or, more broadly, romantic relationships in men’s lives (Allen, 2007; Davies, 2003; Kaufman & Goldscheider, 2007; Nock, 1998), previous studies have largely focused on single women (e.g., Gordon 2003; Budgeon, 2016; Lahad, 2017; Lewis & Moon, 1997; Sharp & Ganon, 2007; Reynolds 2013) and, to a lesser degree, single men (e.g., Apostolou 2019; Davies, 2003; Eck, 2013, 2014; Waehler, 1996). Although men report a weaker “drive to marry” than women (Blakemore et al., 2005), marriage has been closely related to cultural conceptions of masculinity (Kaufman & Goldscheider, 2007) and has even been seen as “a rite of passage into manhood” (Nock, 1998, p. 7). Since the precarious view of the gender status of men (i.e., as a status that requires continual public social proof and validation (Vandello et al., 2008; Bosson & Vandello, 2011), and affects various domains of men’s functioning, including relationships (Bosson & Vandello, 2011), the tenuousness of manhood may require men to establish a new masculinity through romance (Allen, 2007) and taking on the role of a family provider (Davies, 2003).

According to traditional masculinity ideology, men are expected to avoid all things feminine, restrict their emotional life, be tough and aggressive, and have a casual attitude toward sex (Pleck, 1995; Levant, 1996). Stereotypically, men are expected to exhibit greater agency and self-reliance (Ellemers, 2018) and are stereotyped as less empathic, emotionally expressive, and concerned about others (Croft et al., 2015). Therefore, although men may experience less pressure from social networks than women to enter into romantic relationships, cohabitate or get married (Blakemore et al., 2005; Sprecher & Felmlee, 2021), masculine gender norm socialization may influence the way they experience their singlehood. For instance, men may experience singlehood as a way to avoid responsibility and live against a cultural script that expects marriage (Davies, 2003; Eck, 2013, 2014). As men need to constantly resist stereotypical feminine characteristics, traditionally feminine behaviors may generate discomfort. Thus, single men are forced to avoid singlehood scripts stereotypically attributed to single women (Borinca et al., 2021). Single men may also feel that the lack of a romantic partner and romance in their lives hinder the construction of their masculinity.

In previous studies, single men were found to report higher depressive and anxiety symptoms and lower well-being than single women (see Davies 1995, for a review). Never-married men have also been found to be characterized by staunch independence and self-reliance, emotional detachment, interpersonal passivity, and idiosyncratic thinking (Waehler, 1996). Based on these personality traits, three types of never-married men have been distinguished: *flexible* bachelors who are able to drop their independence and successfully relate; *entrenched* bachelors who cling to autonomy to protect themselves; and *conflicted* bachelors who struggle with ambivalence toward staying independent without feeling detached (Waehler, 1996). Single men have also been found to report poor flirting skills, low self-confidence, shyness, and negative experiences from previous relationships as reasons for remaining single (Apostolou, 2019) as well as less fear of being single than women (Sprecher & Felmlee, 2021). Single men have been depicted as womanizers, mama’s boys, nerds, or sexual deviants (Waehler, 1996), as well as irresponsible, self-indulgent, and immature, while married men have been depicted in terms of traditional gender roles such as father, breadwinner, and head of the family (Sakallı Uğurlu et al., 2021).

The research described above has provided several sociological and psychological insights into singlehood among men, but these studies are outdated and may not address the experiences of contemporary single men in the changing context of modern romantic relationships (Bühler & Nikitin, 2020; Mehta et al., 2020) and masculinity (e.g., Christofidou 2021). Moreover, the recent intensive development of singlehood research offers new theoretical propositions and concepts. Specifically, Girme and colleagues (2022) recently recognized the factors that contribute to the within-variability of single individuals without comparing them to coupled individuals and the factors that affect single individuals’ well-being. These factors may include satisfaction with their relationship status (Lehmann et al., 2015; MacDonald & Park, 2022; Park et al., 2021) and fear of being single (Spielmann et al., 2013). Satisfaction with relationship status involves the degree to which individuals are satisfied with their current relationship status (i.e., having or not having a partner/spouse) and is more predictive of well-being than relationship status per se (Lehmann et al., 2015). Fear of being single involves “concern, anxiety, or distress regarding the current or prospective experience of being without a romantic partner” (Spielmann et al., 2013, p. 1049).

In the current qualitative study, we aim to provide insight into the experience of singlehood among contemporary never-married single men living in Poland, a cultural context which is characterized by a strong adherence to heterosexual marriage and weak approval of cohabitation and living single (Janicka & Szymczak, 2019). Poland is also a country where feminist movements have a relatively weak influence on politics and social change, and traditional models of masculinity are strongly promoted (Wojnicka, 2016). Cross-cultural studies show that Polish women and men tend to hold more traditional stereotypes about men, and Polish men are more strongly affected by threats to masculinity than citizens of countries with higher gender equality (Valved et al., 2021). Additionally, anti-gender movements are highly visible in politics and in the public media (Graff & Korolczuk, 2022), and only minor attention is given to men and masculinities in the public discourse (Hearn et al., 2002; Wojnicka 2011). The topic of men’s singlehood remains virtually unaddressed in this cultural context.

## Method

### Study Design

The current paper presents results obtained as part of a larger mixed-method project exploring the phenomenon of singlehood in Poland and its life outcomes. The subject of the experience of singlehood among men emerged during in-depth interviews and the analysis of data collected from a sample of 40 never-married single adults living in Poland. In other words, we retained the openness and flexibility that characterize qualitative research (Słysz & Soroko, 2012) with the data collected in our interviews on the experience of singlehood and its life outcomes. During the data collection, it became evident that single men reported unique experiences of their single status. Thus, we decided to follow this unanticipated research topic.

We grounded the study ontologically and epistemologically in a phenomenological approach to provide an answer to the following question: *How do never-married single men experience their single status?* We chose to describe unique meanings and develop a resonant understanding from the internal (but contextually situated) perspective of single men (Savin-Baden & Major, 2013; Sundler et al., 2019). The study was phenomenologically informed at both the research question formulation stage and the data collection stage (semistructured in-depth interview) as well as during the reflective thematic analysis, which was tailored to the phenomenological approach, thus this paper employed a conceptual rather than a theoretical framework (Miles et al., 2020).

The phenomenological approach allowed us to gather information about the lived experiences of single men in the way they liked to talk about themselves, creating an opportunity (through semi-structured in-depth interviews) to deepen the meanings they make regarding their personal and relational experiences. When conducting the interviews and listening to the stories, the interviewers intended to question pre-understanding; thus, many clarifications and deepening probe questions were made. We used the bracketing interview technique at the interviewers’ preparation stage, which, through the interviewees’ experiences of being interviewed, allows for an exercise in the “epoche” of one’s own experience (Emiliussen et al., 2021). Furthermore, during the reflective thematic analysis, particularly during the coding phase, we emphasized openness in adopting a reflective attitude by, for example, viewing the codes we developed about their biased interpretation and querying whether the codes were sufficiently grounded in the data.

### Participants

Semi-structured in-depth interviews were undertaken with a subsample of 22 single men from a total sample of 40 never-married single adults living in Poland (22 men and 18 women) who were enrolled in a mixed-method project on singlehood among never-married single adults. Considering past literature showing that code saturation may be reached at a smaller number of interviews (e.g., nine) whereas meaning saturation requires a larger number of interviews (e.g., from 16 to 24 interviews) (Hennink et al., 2017), the subsample of 22 men was recognized to be sufficient to provide a broad range of issues in the data and to develop a richly textured understanding of the topics (Hennink et al., 2017).

Participants were eligible if they lived in Poland, were able to read and understand Polish, were in the period of young and established adulthood (i.e., between 20 and 45 years of age when forming an intimate relationship is considered to be a crucial developmental task; Erikson 1980; Mehta et al., 2020), were never-married and had not had a romantic partner/spouse for at least six months, and did not personally know the researchers. The participants ranged in age from 23 to 43 years (*M* = 31.45, *SD* = 5.67). The duration of singlehood ranged from one year to 11 years and included eight participants who identified themselves as always being single. Regarding the desire to have a partner, 13 participants reported a very strong desire to have a partner, six participants reported that having a partner was not a priority life goal, and three participants reported a lack of desire to have a partner and be in a relationship. Twenty-one participants reported a heterosexual orientation, while one reported being homosexual. The basic demographic and relationship status information is shown in Table 1, and detailed characteristics are provided in Table S1 in the supplementary materials at https://osf.io/4ub8t/?view_only=4c44774a6e4a4a28a205f8cdfaf3bf60.

**Table 1:** Demographic and Relationship Status Characteristics of the Participants

	Age	Duration of singlehood (in years)	Desire to have a romantic partner
Participant 1	23	1	Very important
Participant 2	24	always single	Not a priority
Participant 3	25	7	Very important
Participant 4	25	always single	Very important
Participant 5	26	5	Not a priority
Participant 6	28	5	Very important
Participant 7	28	3	No desire
Participant 8	30	1	Not a priority
Participant 9	30	6	Very important
Participant 10	30	6	Not a priority
Participant 11	31	3	Very important
Participant 12	31	always single	Very important
Participant 13	31	always single	Very important
Participant 14	32	11	No desire
Participant 15	32	5.5	Very important
Participant 16	33	always single	Very important
Participant 17	34	1.75	Very important
Participant 18	35	always single	Very important
Participant 19	38	2	Not a priority
Participant 20	40	always single	No desire
Participant 21	43	always single	Very important
Participant 22	43	11	Not a priority

### Interview Schedule

The current investigation employed in-depth semi-structured interviews since they allow for consistency between interviews and flexibility for participants to respond and share their experiences; furthermore, they enable the interviewers to obtain additional clarification with regard to context (Braun & Clarke, 2013). To develop the interview protocol, previous literature on singlehood was considered, and bracketing interviews were employed when the research team (the first, second and fourth authors) were interviewed with the use of the interview schedule to reflect on the genuine experience evoked by the protocol. The final interview protocol consisted of 12 open-ended questions to elicit narratives on topics concerning the experience of singlehood and its life outcomes, which were usually deepened with clarification probes. The narratives were evoked by the initial request, “*Please tell me about your life as a single person [what is it like for you to be single?], including how it used to be, how it is now, and how you see your future*?” The questions focused on the experience of singlehood in terms of thoughts, emotions, and behaviors concerning such major areas as becoming a single person, anticipation of the future in the domain of romantic relationships and single status, congruency between individuals’ romantic desires and current single status, uncertainty and feelings with regard to finding a partner in the future, and perceived outcomes of singlehood on daily functioning and the impact of the COVID-19 pandemic on single living. The entire interview schedule is provided in the online supplementary materials, available at https://osf.io/4ub8t/?view_only=4c44774a6e4a4a28a205f8cdfaf3bf60.

### Procedure

The investigation was preceded by obtaining approval from the Research Ethics Committee at the Faculty of Psychology and Cognitive Sciences, Adam Mickiewicz University in Poznań, Poland (Decision number: 2/07/2020). After approval from the institutional ethics review board, purposeful recruitment was conducted via advertisements placed on the Facebook website affiliated with the current project. After clicking the link, participants were directed to a survey where they were provided with detailed information concerning the study’s aim and procedure and an opportunity to leave their contact details and availability for an interview. Participants were contacted to confirm their eligibility and time to attend the interview. Basic demographic information and informed consent were gathered prior to the main interview schedule.

The data were collected between December 2020 and May 2021. Due to the COVID-19 pandemic and social distancing restrictions, similar to the study by Williamson and colleagues (2023), interviews were conducted online and were audio-recorded. The duration of the interviews ranged from 28 to 95 min (*M* = 62 min). The same set of questions was presented to each interviewee with the necessary prompts. At the end of the interview, participants had the opportunity to raise any concerns about their participation in the interview. All interviews were transcribed verbatim, and participants were assigned a unique code to protect their anonymity. All participants received compensation for their participation in the form of vouchers to an online bookstore with a value of 90 PLN.

### Data Analysis

The data were analyzed by employing reflexive thematic analysis, an inductive technique of identifying and interpreting patterns in qualitative data (Braun & Clarke, 2021a, b). In the analysis, we were led by a phenomenological approach. We applied a rich description of data aimed at identifying recurring patterns of meanings based on detailed and systematic (line-by-line) inductive coding with a focus on both explicit (semantic) and implicit (interpretive) levels of content. This dual-level solution enabled us to grasp both overt declarations and covert perceptions, attitudes, and feelings to form accurate and vivid interpretations of patterns of meaning (themes) across the dataset. In line with reflexive analysis (Braun & Clarke, 2021a, b), we did not use a coding framework or calculate the coders’ agreement, but we developed codes and derived themes in an iterative, recursive manner with a reflective stance.

During the analysis, we followed the steps proposed in the classic article by Braun and Clarke (2006) enriched by the guidelines articulated by these authors in recent works (Clarke & Braun, 2016). The steps followed include: (1) familiarization with the data, (2) coding, (3) searching for themes, (4) reviewing themes, (5) defining and naming themes, and (6) writing up. An additional element was performing the analysis as a team to ensure quality control in the form of investigator triangulation and adopting a reflective attitude toward our data and analytic process. We benefited from engaging three different investigators (the first, second, and third authors) to analyze the material and to compare the process of coding and creating themes, what each researcher highlighted, and what he or she missed (Denzin, 1978; Nowell et al., 2017). The fourth author provided an audit by reviewing the themes, giving feedback on their comprehensibility, communicability, and clarity, and helping to organize them into a meaningful whole.

Through discussion, all authors obtained final team consensus on the themes, which we present in the article. Consistent with phenomenological sensitivity, working in a team allowed us to explore each of our multiple assumptions during the coding and to capture possible biases arising from the difficulty of capturing our own preunderstanding. Indeed, spotting potential biases in each other was valuable. Working on a common list of themes as a team also allowed us to capture more meaning and select miscellaneous quotations. A detailed description of the step-by-step process of analysis and details regarding establishing the trustworthiness of the current investigation are provided in the supplementary materials, available at https://osf.io/4ub8t/?view_only=4c44774a6e4a4a28a205f8cdfaf3bf60.

### Researcher Reflexivity

To determine reflexivity and acknowledge and capitalize on our subjectivity, in the current investigation, we have identified and reflected on our social identity (Jacobson & Mustafa, 2019; Olmos-Vega et al., 2022). We identified with our metric sex (three women, one man). Each researcher was in a long-term relationship and had at least one child; however, they had experienced singlehood in the past, albeit not in an acute form. Most of the interviews were conducted by women (the first author conducted six interviews and the fourth author conducted nine interviews), while the second author conducted seven interviews, contributing to the potential variation in the data in terms of revealing experiences due to the listener’s gender. All the authors were psychologists specializing in different fields: the first author in clinical psychology, psychotherapy and sociology, the second author in identity research and the role of future thinking for emerging adults’ development, the third author in qualitative methodology and clinical psychology, and the fourth author in the field of singlehood research and romantic relationships. In addition, two of them (the first and the third authors) had practical experience in diagnosis and psychotherapy, which influenced their balance between deep empathy and neutrality during the interviews and analysis. Thus, our research was psychologically informed in at least two ways: (1) we were interested in how our participants thought, felt, and made meaning of their singlehood, and we decreased arbitrary judgments or directiveness, and (2) we constructed themes in terms of psychological activities people perform to understand themselves. The team working on the thematic analysis (the first, second and third authors) did not intentionally activate psychological theories of singlehood (apart from general knowledge of the research project). Moreover, we commented on potential biases in understanding men’s narratives (during the phase of meetings comparing coding effects). The fourth author acted as a supervisor for this research, adding theoretical polishing to the description and discussion of the findings.

## Results

We present the results of the thematic analysis below. Five key themes were identified that reflect the nature of singlehood as experienced by never-married single men. Table 2 provides a summary of these themes.

**Table 2:** Summary of Themes, Definitions, Example Quotes, and Frequencies

Theme	Description	Example quotes	Frequency *n* (%)
The sense of being deficient: is there something wrong with me?	Single men have a sense of having various deficits (e.g., medical conditions, psychological or family factors) that hinder or completely exclude the possibility of finding a romantic partner and establishing and maintaining a satisfying, long-term romantic relationship. Single men differ in their recognition of the depth of these deficits and their capability to change them.	Until recently, I was avoiding friendships… These problems were related to toxic relationships in my family of origin. In my family, in case of any problem, my reaction was to distance, to cut off. And this cutting off became second nature to me. As a result, I tend to avoid any relationships with other people that are more serious relationships. Only now it has started to be better thanks to psychotherapy. (Participant 12)	18 (82%)
Navigating outside the dominant discourse of traditional masculinity, marriage and family	Single men search for their own way beyond the mainstream and dominant discourse pertaining marriage and family. They also renegotiate their personal masculinity and search for a way to be a single man in a society devoted to traditional gender roles and the high value given to traditional marriage and family.	Our society knows the meaningful formula mostly in family life. (Participant 22) I have been trying to meet these [traditional masculinity] expectations, but paradoxically, I’ve started meeting women who wanted something different, and I was confused. (Participant 5)	18 (82%)
Benefits and the downsides of singlehood	Men experience (often simultaneously) singlehood as involving both advantages and disadvantages. As the most common benefit of single status, men report independence, time and energy for occupational and personal development, time for a hobby, and a lack of problems typical of couples and parents. Among the most frequent downsides of single status, men indicate various negative emotions (e.g., sadness, anger, emptiness, grief), isolation, loneliness, lack of meaning in life, and unsatisfied needs (emotional, social, sexual).	I can’t deny that being single is convenient. It is somehow convenient. It is not necessary to worry about the second person. There are pros and cons. (Participant 2) Sometimes, when I have experienced difficult situations, my mood tends to decrease… and I wanted someone to hug me… That was kind of emotional suffering. (Participant 12)	20 (91%)
Adaptation to singlehood	Single men employ various strategies to adapt to their singlehood. This involves adaptive strategies (e.g., acceptance, investing in personal development) and maladaptive strategies (e.g., isolation, emotional distancing, compensative activities, substance and behavioral addictions).	I am ok with that who I am and where I am in my life now. I think I prefer to preserve that state rather than being with someone at a push. However, I plan to be in a romantic relationship in the future, and I strive for that. (Participant 16) When dark thoughts come, uncertainty, considering what the problem is - maybe my physical attributes… weight […] When these thoughts are too intrusive, I assuage them with alcohol. Not drugs, but alcohol. After drinking a larger amount of alcohol, there is at least temporary peace. I can read a book, and these thoughts don’t come to me - “why am I lonely? Will I meet someone this year? (Participant 15)	21(96%)
Dilemma between waiting and actively searching for a romantic partner	Single men experience the dilemma of whether they should wait until a romantic partner appears or whether they should actively search for a partner, for instance, by dating, improving communication skills, or searching for a possibility to meet a potential partner. This dilemma reflects the tension between passively waiting for a partner, which is considered by men to be a more feminine strategy, and undertaking actions to find a partner, which is considered a more masculine strategy.	I think I’m waiting. I am just a bit scared of that inertia in general. The waiting, that if I sit, let’s say, and wait, not knowing for what, that person might not come. […] It’s now more difficult, due to this pandemics, actually everything is closed. […] Everything has gone online, so it’s more difficult, more difficult to get to know someone. At first, there were even some speed dates; signing up was possible. I signed up; but after a week, it turned out they would not happen, they wouldn’t, so […] I am a bit afraid of that. So not wait but try, at least a little bit. (Participant 15)	17 (77%)

### The Sense of Being Deficient: Is There Something Wrong with Me?

Many of the interviewees (*n* = 18, 82%) reported a sense of possessing some deficits that hindered their possibilities of finding a romantic partner and establishing a serious, long-term relationship. The sense of being deficient was often accompanied by the recurring question of whether there was something wrong with them because they were single. Some men believed that they were not in a relationship due to serious medical conditions or psychological disorders (e.g., infectious disease, depression, alcoholism, autism). The power and indisputability of illness as a barrier that permanently stood in the way of finding a romantic partner and establishing a relationship was emphasized by a 40-year-old man who was infected with hepatitis: “Any contact with me is dangerous (…). This is a potentially lethal disease, incurable at the moment” (P20).

Additionally, psychological and developmental disorders were considered to impair the social skills needed to find a romantic partner and maintain a relationship. A 34-year-old man who was diagnosed with autism spectrum symptoms said:

Because of this Asperger’s I think I could have been acting a bit out of line. For example, when I found out [while on a date] that her grandfather died in a cinema, I could not stop myself from smiling (P17).

Some single men reported negative experiences in their family of origin and.

had the sense that these past experiences currently hindered their possibility of being involved in a serious romantic relationship and contributed to withdrawal and/or a feeling of inadequacy in the domain of romantic relationships. For instance, a 31-year-old man said, “There was a problem of psychological abuse in my family. I developed mechanisms so that I could adapt to this kind of situation (…). These mechanisms resulted in avoiding any kind of relationship (…)” (P12). A 32-year-old man viewed his father as a negative figure who contributed to his feeling of insecurity in the domain of close relationships:

Actually, I didn’t have a happy family because my father took out all the frustrations derived from his childhood on my mother, me, and my brother. And this is maybe such a problem (…) because I am actually quite afraid of all interpersonal relationships (P15).

Some men were able to articulate the sense that they could “fix themselves” with enough effort, practice, and/or professional help. Some participants had been or currently were in a long-term therapy process, which they believed helped them to identify and solve the issues related to the domain of romantic relationships. For instance, a 26-year-old man described the role of psychotherapy in his relational struggles:

I met a girl [...] who was attractive, [...] I went for a cup of coffee with her and [...] I almost felt asleep there. [...] When I was talking it over with my therapist, we both noticed that she was very similar to my mum. [...] It was a huge [...] “AHA! Oh my God, this is why it’s all happening this way” moment for me. [...] I would not have noticed it if not for the therapy” (P5).

Other men employed more technical solutions, such as improving their self-image and learning social skills needed in the domain of romantic relationships. A 35-year-old single man who strongly desired to be in a relationship noted the need to improve his relational skills by engaging professional help:

That is, I went to a lady who works as a love coach [an academically trained psychologist and sexologist] and just, like, with her help I’m trying to work on my image and on what I can do to even open myself to a relationship, and, in the long term, to find a partner (P18).

At the same time, some men felt that it would be very hard or impossible for them to change and therefore considered avoiding close romantic relationships and withdrawing from starting a family. A 28-year-old man noted, “I have some problems and I am no longer able to change them, and let’s say they have been around for 10 years. I perceive that they have always existed and exist now” (P7).

As demonstrated, single men often experience a sense of being deficient in regard to various aspects of their lives. They consider their personal deficits to be the causes of difficulties in finding a romantic partner, establishing a romantic relationship, and subsequently starting a family. In addition, they consider these deficits to range from profound and unchangeable to challenging but repairable to small skill gaps that could be easily remedied. The perception of deficits as profound and unrepairable often results in the feeling of resignation and the abandonment of relational goals. In contrast, the perception of deficits and problems as less severe enhances the sense that these deficits may be altered even if overcoming these deficits requires greater personal effort, including the will and readiness to use professional psychotherapy, which is already utilized by some participants.

### Navigating Outside the Dominant Discourse of Traditional Masculinity, Marriage and Family

Many of the men interviewed (*n* = 18, 82%) reported feeling different and isolated due to their single status. They expressed the sense that they missed out on something important and that they fell behind on the social schedule, meaning getting married and starting a family at some point in life. A 35-year-old man noted that the sense of “being off time” often resulted from comparing himself as a single person with others who are coupled:

My sister got married last year (...), my brother (...) has a girlfriend (...). I am the oldest and I feel like I should be the first [in a relationship] (...) while it turns out that I probably will be the last one to engage in a relationship, and it really bothers me (P18).

Some participants expressed concerns regarding the “biological clock” and whether they would be able to start a family by the time they were 40. They claimed that late parenthood was risky because it increases the probability of genetically based diseases and that after 40, one is too old to be a proper father. A 43-year-old man who strongly wanted to have a romantic partner noted with sadness, “I would still like to start a family someday, well, but we know that at my age it can already be difficult with children” (P21).

At the same time, the recognition that one lives outside of the dominant pattern did not necessarily imply the urge to change this. In contrast, relief related to avoiding getting married and having children was sometimes reported. However, being single implied the need to find one’s own formula for a “meaningful life”, as expressed by a 43-year-old man:

All my life I believed that this was the preparatory stage and that this proper life would begin with someone, in a relationship. And now I face this truth that maybe there is no next stage (...) and I must find a good way to live a single life (P22).

Furthermore, single men recognized how they differed from the dominant script of traditional masculinity and gender roles. For instance, they struggled with the stereotypical view of a single man as a womanizer and highlighted that they looked for serious romantic relationships, not casual sex. A 38-year-old man noted, “Many guys are there [on Tinder] just to get straight to the point (…). I cannot imagine myself how I could meet someone (…) and start proposing sex” (P19).

A 26-year-old man stated that he lacked the ability to fit into the dominant pattern as the cause of remaining single and that rejecting dominant patterns was necessary for him to stay in harmony with himself:

I often hear that I am too ‘warm’, (…) not a ‘typical man’, rather a ‘teddy bear’. And that wasn’t interesting for the women I admired and wanted. I tried to do something for myself, something that would be a compromise (…) something that would be more in line with the expectations of women [being a heavily built, dominant man] and, at the same time, would be good for me’ (P5).

In summary, single men experience several challenges resulting from navigating their lives outside of the couple-oriented discourse, away from the traditional masculinity and gender roles present in Polish society. Single men feel that they need to respond to the pressure coming from the prevailing societal scripts and the ideology of marriage and family; they also need to face the fear of being single, and, while doing all this, keep trying to remain in harmony with themselves, maintaining the pursuit of their own goals.

### Benefits and Downsides of Singlehood

Nearly all the men (*n* = 20, 91%) emphasized that single status has advantages and disadvantages. The benefits of singlehood were mainly related to independence, autonomous decision-making, and being able to focus on personal development, pleasures, and hobbies. For instance, a 38-year-old man indicated self-centering and self-determination as two benefits offered by single status:

I alone make the decisions, and I am accountable to no one but myself (…). A relationship is really the art of daily adjustments and compromises (…), and I don’t have what it takes (P19).

The downsides of singlehood were predominantly related to experiencing singlehood as difficult, concentrating on the lack of a partner, unmet relational needs, and loneliness. This overwhelming sense of loneliness was, for instance, reported by a 31-year-old man for whom finding a partner was extremely important: “Sometimes I start crying because of this loneliness (…). I just feel bad, I feel lonely, I feel like something is missing, I miss something and I am not satisfied, I feel hungry” (P11). The negativity of remaining single was sometimes related to the perception of the lack of a romantic relationship as a cause of low self-esteem. A 32-year-old man noted, “There are days when I feel like a total loser, that I have lost my life because I am not part of a relationship, I am not part of a family” (P14).

Single men also talked about the unmet needs for emotional, physical, and sexual intimacy because of remaining single. These experiences were vividly described by a 28-year-old man who strongly desired to be in romantic relationships: “I would like to share my time with someone, to live together, to share some of my interests, you know, to have a sexual life, to have someone for psychological support, to share my inner life” (P6).

For some men, being single also meant the loss of the possibility of becoming a father. This experience was even reported by a participant who did not desire to be in a romantic relationship. A 40-year-old man admitted, “To be honest, the fact that I don’t have my own children is my biggest failure (…). This is something I regret the most” (P20).

Finally, the experience of singlehood in terms of both advantages and disadvantages often confronted our participants with mixed emotions, conflicts, and questions regarding whether they should try to find someone or whether it would be better if they just accepted their single status. This experience was reported by a 33-year-old man who said, “I got used to it because this self-reliance is comfortable (…) Although I think it’s a bit of a waste to have this life solely for yourself” (P16).

In summary, men do not consider singlehood in zero-one terms. In contrast, they have a sense that singlehood simultaneously involves many positive and negative aspects. The recognition that singlehood involves both bright and dark sides makes the experience of singlehood a source of ambiguity and conflicting feelings contributing to an ambivalent attitude. The perceived advantages allow single men to positively construct their experience of singlehood, whereas the perceived disadvantages diminish satisfaction with the single status.

### Adaptation to Singlehood

In addition to recognizing the advantages and disadvantages of single status, almost all single men (*n* = 21, 96%) recounted how they adapted to the situation of remaining single. For some of them, stoic acceptance and trying to make the most of the challenging situation was a basic strategy, as in the case of a 30-year-old man who recognized that focusing on his career may be a way to manage single status:

Whatever happens will be OK with me. If I don’t meet someone (…), so be it. Then I will devote that energy, which I would otherwise spend on someone, to improving, simply improving the people around me, OK? That is, I will devote myself more to the club [his work], I will devote more to helping the needed, yes. So yes, these are generally my plans (P10).

Other participants used strategies based on avoiding negative feelings arising from being single. A 31-year-old man reported, “I just disconnect from emotions that are difficult for me. And this is one of the reasons why I better cope with the fact that I am alone at the moment” (P12).

Men in the current study also used various activities to help them divert attention away from their singlehood, as in the case of a 25-year-old who strongly wanted to find a partner:

I need to be involved in an activity because my thoughts sink in irritation when I lay back and allow myself any period of inactivity. […] As long as I have training and activities and I am acting, it is not bad (P3).

Sometimes the participants employed less constructive coping strategies, such as excessive eating, drinking, or gaming and pornography overuse. A 30-year-old man vividly described this: “In order not to feel all those things, I escaped by eating, drinking (…), using the computer excessively (…). I escaped to the virtual world not to be alone” (P10).

To summarize, most single men differ in their abilities to confront their emotions related to being single, competencies needed to cope with romantic rejection and being alone, and in the ways in which they organize their single lives. Therefore, single men employ various strategies to adapt to and be satisfied with their single status and to lead the most authentic and meaningful life. Some of these strategies constitute constructive strategies (e.g., embracing singlehood or investment in personal development or career), allowing them to preserve happiness and satisfaction as a single person. However, some strategies represent less adaptive means to cope with singlehood and include destructive behaviors such as alcohol overuse or distancing from emotions.

### Dilemma Between Waiting and Actively Searching for a Romantic Partner

Many participants (*n* = 17, 77%) talked about the experience of an internal discourse with themselves about whether they should wait for the right partner or take active efforts to find someone. These deliberations reflected being passive and being activite as polar opposites when searching for a partner.

The passive attitude mostly meant waiting for a partner and was vividly illustrated in the statement of a 31-year-old: “I’m waiting all the time (…). Maybe it sounds weird (…) but I just wait for my princess on a white horse” (P11). A 25-year-old man had been waiting for an impulse to act that “would encourage me to write something [on online app], knowing that someone made the first step and I can follow, act” (P3).

Being passive also served for some men as a way to protect themselves from painful rejections. It is illustrated by this statement of a 32-year-old man who was single for over five years:

I had [...] high anxiety [...] and I often even really wanted, really wished to behave more confidently, to say something in the presence of a female friend [...] or, I don’t know, to bring about a situation, where [...] at a party I could take her aside and talk [...], establish some contact [...] but [...] I lost that chance, because I was fearing [...] the judgment too much, I was fearing rejection (P15).

While some participants had been waiting for a romantic partner to appear, other men wanted to be active and retain control in searching for the right partner. This proactive approach was demonstrated by a 33-year-old always single man for whom finding a partner was very important and who had a strong sense of internal locus of control: “I definitely don’t wait for something to happen or change by fate. Life is what you make it. The direction of my life depends only on me… and this second person, yes” (P16).

This proactive approach to finding a romantic partner was recognized by our participants as requiring various strategies, such as working on their own personal deficits (including psychotherapy), establishing financial independence, developing communicative and dating strategies, starting or polishing existing accounts on dating apps, and visiting places where they could meet potential partners. This need to employ diverse means to alter single status was described by a 25-year-old man:

I want to intensify my social life, meet people and visit places where it is possible to meet someone (...). It is not impossible that I will try, as an experiment, to register on a dating website (...). Maybe I will go back to singing… I want to meet people and check if I can still fall in love. Maybe I will meet someone nice. At the same time, get my personal life together: psychotherapy, physical activity, taking care of my health (P4).

The choice of between being active or passive appeared to be influenced by the participants’ beliefs in regard to the appropriate strategy to find someone. Some men believed that they needed to do something to change their single status. A 24 year-old, always single man thoroughly described his conflict between personal preference to wait for the right partner and the belief that one needs to be active in order to find someone:

I expect that long waiting will allow me to live to see the day. On the other hand, I have some presumptions that sometimes, or even usually, it is necessary to take matters into one’s own hands and look for someone. I would prefer it if it worked like that, like it is enough to wait sufficiently long to find someone, but it is not an appropriate attitude from the perspective of me as a teacher, coach, and educator (P2).

In turn, other men believed that fate decides if their patience and efforts will be rewarded by finding true love. This view was vividly described by a 35-year old, always single:

It may be claimed that I am waiting for this person. I still believe, somewhat biblically, that “It is not good that man should be alone” and that somewhere there should be this one person intended for me. [...] So, it is that kind of thinking - she is somewhere, I need to be patient, and someday my effort and endeavor will be awarded by finding this person (P18).

The “waiting or acting” dilemma was related to negotiating the traditional pattern of masculinity, which implies that men should have control over their romantic and sex life. A 32-year-old man who held a more traditional view on which gender should be active in the romantic domain noted, “It is not my role to wait. I, as a man, I am the active party, and it is on me to possess the initiative to start any kind of relationship” (P14).

As demonstrated, single men hold different attitudes toward the issue of searching for a romantic partner and how single status could be altered. Single men recognize that approaches to searching for a romantic partner are located on a continuum ranging from passivity and waiting to actively seeking a partner, meaning undertaking various actions to find the right lifetime partner. Some of them prefer to be active and decline passivity and waiting for a partner. The participants acknowledge differences in the perceived locus of control in the domain of romantic life and the real possibilities to change their single status when it is experienced as involuntary and unsatisfying. These two approaches strongly connect with men’s ideas about masculinity and perceived social expectations toward men and reflect the dilemma concerning the choice between passively waiting for a partner, which is considered a more feminine strategy, and undertaking actions to find a partner, which is considered a more masculine strategy.

## Discussion

Our goal in the present qualitative study was to shed light on Polish never-married single men’s experiences of singlehood. In line with the recently postulated need to apply the within-group perspective on singlehood and recognize the diversity of single individuals’ experiences (Girme et al., 2022), the current study demonstrated that singlehood represents a challenge for men and entails the need for establishing one’s identity in reference to the single status, also including the necessity to reconsider masculinity and undertake various actions to earn it (Bosson & Vandello, 2011) in the context of singlehood. The diverse, complex, and dynamic experiences of 22 single men were reflected in the current investigation in the five themes identified in the analysis: (1) the sense of being deficient: is there something wrong with me?; (2) navigating outside the dominant discourse of traditional masculinity, marriage and family; (3) the benefits and downsides of singlehood; (4) adaptation to singlehood; and (5) the dilemma between waiting and actively searching for a romantic partner.

## The Sense of Being Deficient: Is There Something Wrong with Me?

Consistent with previous literature (Apostolou, 2019; Bergström et al., 2019; Lewis & Moon, 1997; Reynolds, 2013), the first theme reflected a sense of possessing various deficits involving medical and psychological conditions that were perceived as obstacles to finding a romantic partner and establishing a serious, long-term romantic relationship. For instance, past studies have shown that being single is treated as a display of “disability,” a “hidden defect,” or a “flaw” (Bergström et al., 2019), and as a failure to establish a relationship (Apostolou, 2019). These experiences reflect some aspects of a fear of being single, which includes the sense that failure in finding a partner means that there is something wrong with a person (Spielmann et al., 2013).

In addition, our study showed that one of the important motifs in the collected narratives was the notion that dysfunctional ties in the family of origin and a negative or absent father figure had a detrimental influence on men’s ability to establish a satisfactory long-term romantic relationship. A similar reference to experiences in the family of origin was found in a study by Czernecka (2016), in which a negative image of the relationship between parents was perceived as obstructive in the formation of relationships by never-married single men and women aged 25–40 years old. Our findings therefore support previous studies showing a positive association between the quality of relationships in the family of origin and future romantic partnerships (Walper & Wendt, 2015), and the link between poor family relationships and subsequent singlehood among men (Waehler, 1995).

Importantly, the narratives concerning various deficits also involved the perception of how deep and profound their problems in establishing a relationship were. Some men felt the need to change themselves to find a partner and be a good partner in the future. If men perceived this change as impossible or extremely difficult, such as in cases of incurable infectious diseases, mental disorders, addiction, or the autism spectrum, they were likely to feel excluded from the relationship domain. However, if there was a chance to improve themselves and the desire to have a romantic partner was strong, some men were willing to start or had already started working on themselves, including professional help. Moreover, they did not express the feeling of being stigmatized by the need for psychological intervention and sincerely hoped that it would help them fulfill their relational needs. This finding contradicts past studies suggesting that men tend to avoid psychotherapy (Brooks, 2017) and the gender stereotypes and traditional masculinity scripts that imply self-sufficiency, emotional restraint, and a focus on success and power (Mahalik et al., 2003; Levant et al., 2022).

### Navigating Outside the Dominant Discourse of Traditional Masculinity, Marriage and Family

The second theme revealed that the men viewed their single status as leading them away from the traditional life path involving marriage and family (Budgeon, 2016; DePaulo, 2006; DePaulo & Morris, 2005; Morris et al., 2008) and from the prevailing narratives of traditional masculinity and marriage (Kaufman & Goldscheider, 2007). Some men thought they were single because they could not fit traditional masculinity norms, such as being emotionally tough and physically strong. Some believed that a man needs to be capable of providing for a family if he wants to establish a long-term relationship (Kluczyńska, 2009; Wong et al., 2020).

The single status evoked in our participants the feeling of falling behind the socially expected life schedule, stagnating in one’s personal development, and being isolated, excluded, and pressured to find someone. Indeed, this feeling of “being off time” and not achieving important developmental milestones in the domain of romantic relationships and family often derived, as shown in previous studies, from comparing oneself as a single person with peers who were already coupled (Donnelly et al., 2001).

Our findings extend previous work demonstrating that the sense of being outside of the mainstream ideology of marriage and family also relates to an adherence to traditional masculinity norms, including the expectation that men should be sexually active (Endendijk et al., 2020). Our participants struggled with negative stereotypes about single men and pressure from traditional masculinity ideology and gender stereotypes (Ellemers, 2018; Levant, 2011) as the conceptions of traditional masculinity include being married (Kaufman & Goldscheider, 2007) or having a romantic relationship (Allen, 2007).

Some men openly worried that because of their singlehood, they would be perceived as womanizers because single status has been considered to enable individuals to engage in casual sex (Eck, 2014; Waehler, 1996). Our findings imply that some single men perceive sex as an inherent element of a romantic relationship and therefore are not interested in merely casual sexual relationships. Contrary to prevailing stereotypes, the single men in our study strongly resisted playboy norms, which emphasize a desire for frequent sexual activity and partners (Mahalik et al., 2003; Wong et al., 2020).

Though men are generally more likely to engage in casual sex (Pipitone et al., 2021) than women, and masculine norms include the expectation that men be sexually active (Endendijk et al., 2020), the results of our study demonstrate that some men do not follow these gendered behaviors. The men in this sample emphasized that although they sometimes suffered from the lack of a sexual life, they would not regard one-night stands as enjoyable, and they valued intimacy more. These men might therefore be described as involuntarily celibate, i.e., as persons who desire to have sex but are unable to find a willing partner (Donnelly et al., 2001) with whom they could be in a romantic relationship. The case of involuntary celibacy among single men may be an interesting direction for future research.

Importantly, our study uniquely showed that single men experience not only a loss of intimate relationships but that their single status is also a key obstacle for achieving other important developmental and life events, such as becoming a father. Becoming a father can be an important source of happiness and fulfillment for men (Baldwin et al., 2018; Nyström & Öhrling, 2004). Specifically, some men expressed concerns that it may be too late for them to find a partner and start a family, which reflects one of the aspects of the fear of being single (Spielmann et al., 2013), and hinders the achievement of satisfaction with single status. Furthermore, as Eck (2013) noted, being single may confront men with an unanticipated loss of identity as a father which is expected to be established in line with social and cultural norms legitimizing the masculine status (Borgkvist et al., 2020). On the other hand, some participants emphasized that one of the benefits of single status is the lack of responsibilities and burdens related to fatherhood. The men’s narratives indicated that men may also experience parenthood as related to various difficulties, fears, negative emotions, stress, and limitations (Baldwin et al., 2018; Nyström & Öhrling, 2004), and that remaining single liberates them from these negative aspects of fatherhood.

### Benefits and Downsides of Singlehood

The third theme further revealed the men’s ambivalent feelings evoked by being single. Some of the men highlighted several benefits associated with singlehood, such as autonomy, self-determination, and the lack of responsibilities associated with providing and caring for the family. These positive aspects of singlehood align with the recognition that some single individuals highly value independence and have less desire to affiliate with others (Park et al., 2023). In addition, these values appear to reflect the view of traditional masculinity as related to greater agency and self-reliance (Ellemers, 2018) and suggest that for men, being in a relationship means taking on a role as a family provider; therefore, singlehood allows men to avoid this responsibility (Davies, 2003). These positive aspects of singlehood contribute to greater satisfaction with single status and provide the possibility to embrace singlehood through the positive construction of singlehood (Reynolds & Wetherell, 2003). On the other hand, single men in our study described an unmet need for intimacy (i.e., incongruency between the strong desire to have a partner and single status) and regret about not having a family and children. Since romantic and intimacy needs are important for men (Korobov & Thorne, 2006) and romance may be an essential element of masculinity (Allen, 2007), the lack of physical and emotional intimacy was experienced by our participants as one of the most painful downsides of single status. In turn, this unmet desire to have a partner and lack of intimate relationships diminished their satisfaction with their relationship status and exaggerated their fear of being single, reflected in their fear of living alone for their whole life (Spielmann et al., 2013).

This acknowledgment of both positive and negative outcomes of singlehood as well as ambivalence toward single status is consistent with earlier papers (Darrington et al., 2005; Jackson, 2018; Lewis & Moon, 1997; Palus, 2010; Reynolds, 2002). The experiences of some men also resembled the experiences of Waehler’s (1996) conflicted bachelors who struggled with the ambivalence between remaining independent and feeling detached. Analogously to single women in the study by Reynolds and Wetherell (2003), singlehood was a troubled category for men in our study and confronted them with mixed emotions. At the same time, our findings uniquely demonstrate that single men can acknowledge the ambivalence of singlehood and, in some cases, tolerating it.

### Adaptation to Singlehood

The fourth theme revealed that men are able to adapt and adjust to single status. Participants from the current study reported that they coped with singlehood by avoiding negative feelings or adopting stoicism as a basic strategy. Men also coped with single status through work and other achievements. At the same time, the present narratives uniquely reveal that single men sometimes employ problematic coping strategies such as excessive eating, drinking, gaming, and pornography overuse. Therefore, the way men cope with singlehood appears to draw from traditional masculinity norms of coping, involving strategies such as emotional restraint and stoicism (Jansz, 2000), the tendency to use emotional inhibition and suppressive strategies (Matud, 2004), and the likelihood of substance abuse to deal with stress (Rosenfield & Mouzon, 2013). The possible scripts available to men for coping with the downsides of singlehood are limited. Various scholars have pointed out that adapting to singlehood is difficult because it is socially recognized as an unnatural and temporary condition, while establishing a long-term relationship is seen as the most appropriate solution (Morris et al., 2008; Reynolds & Wetherell, 2003; Lahad, 2017). Apparently, these scripts may be even more limited for men.

### Dilemma Between Waiting and Actively Searching for a Romantic Partner

The fifth theme demonstrated the choice that men considered between waiting and actively searching for a romantic partner. Some of our participants preferred passive waiting, hoping that they would be noticed and chosen by a potential partner, which reflects the concept of interpersonal passivity identified in single men in past work (Waehler, 1996). This theme reveals how following the script of an active dominant man may be emotionally difficult. For some of our participants, their passiveness was a way to defend their well-being as they experienced difficulties and anxieties regarding rejections from potential partners. Men who attempted to be proactive in finding a romantic partner described taking the initiative, developing personal and dating skills, working on personal deficits, and preparing for possible challenges related to dating.

Overall, the participants experienced a tension between passivity and being proactive. In some cases, the participants believed that as men they needed to be proactive (Eaton & Rose, 2011), and they had doubts regarding their masculinity if they tended to withdraw. This proactive approach may help to affirm manhood status as a single man, which, in accordance with the concept of precarious manhood, must be earned and demonstrated repeatedly through actions (Bosson & Vandello, 2011). It should also be noted that the men in our study held different views regarding the locus of control in finding true love; some believed that it depended partly on fate, which decides whether they would eventually meet someone, while others were certain that it was up to them and their actions. Like the men in Hartman’s (2017) study who resisted socially imposed definitions of their subjective experience of a partner-initiated breakup and used strategies ranging from resistance to accommodation, some men in the current study also felt the need to negotiate with masculinity norms to stay in harmony with their individual experience of singlehood, including the various preferred ways of pursuing a romantic partner.

### Limitations and Future Research Directions

Although the current study has provided important insight into how never-married single men experience their singlehood, it should also be considered in the context of its limitations. The aim of this study was not to provide generalizable outcomes for all single men but to provide a description of the experiences of never-married single men living in Poland during the period of young and established adulthood. The experiences of our participants may not reflect the experiences of other groups of single men (e.g., divorced or widowed) and men from other age groups living in non-Polish marital and relational contexts. Future research would benefit from exploring the experience of single men of various relational histories, in the period of middle and old adulthood, and originating from other marital and relational contexts. In addition, our sample consisted predominantly of heterosexual men and greater inclusion of non-heterosexual men is needed to investigate the nature of their experiences with single status, which may differ from the experiences of heterosexual men (Hostetler, 2009).

Our participants were recruited via Facebook, which implies a self-selection bias. It is possible that our recruitment strategy might have resulted in a sample of men who were particularly interested in our research question, for instance, men who either felt distress due to their single status or, on the contrary, felt comfortable being single and were willing to talk about it in an interview. It could also attract men who were particularly open and reflective in analyzing their attitudes and feelings. It is also possible that men who adhere to traditional masculine norms may avoid participating in psychological research, especially knowing that it would include talking about their personal relationship experiences. Future research might consider employing more targeted recruitment strategies than social media.

The interviews in the current investigation were performed by a mixed-gender research team consisting of one male and two female researchers. Regarding the discussion in the literature with respect to the role of gender in constructing the context during in-depth interviews, and different behaviors of the interviewees in same- or mixed-gender interviews (Williams & Heikes, 1993), it cannot be ruled out that this mixed-gender composition of the research team might have evoked particular presentations and stories provided by our single interviewees.

Finally, due to the occurrence of the COVID-19 pandemic, our interviews were conducted online, which is likely to affect the dynamic between participants and interviewers. Further and most importantly, the negative impact of the COVID-19 pandemic on mental health and feelings of isolation in particular (Carotta et al., 2022) may have contributed to a greater salience of negative experiences for the men to draw upon during the interviews. Therefore, it is possible that interviews conducted pre-COVID-19 pandemic and/or in-person interviews may have revealed different experiences of singlehood for participants.

### Practice Implications

The findings of this investigation have several implications for practice. First, the results contradict the view that single status is problematic only for women. Our study shows that single status may impact men’s self-esteem, satisfaction with life and single status, and sense of personal development and life fulfillment. Psychotherapists, counselors, and educators working with single men may need to address their single status and its emotional consequences to help them improve and preserve their mental health. Mental health professionals may also help single men who want to be in a romantic relationship identify the objective obstacles that can be dealt with and propose targeted interventions such as therapy or social skills training. They may also help single men deal with the feeling of being deficient and disabled by showing how this feeling may be partially generated by negative stereotypes regarding single men.

Our results suggest that specialists working with single men could help them by recognizing how difficult it is for them to fit into the mainstream discourse dominated by traditional masculinity and couple ideology because this discourse is too narrow to contain the richness and complexity of their personal experiences. They can help their male clients and patients deal with how their single status puts them on an alternative life path that lacks some of the traditional developmental milestones for men, leaving them without a proper script for achieving personal and emotional growth. Professionals should also be attentive to how traditional masculinity ideology labels single men as playboys or womanizers and should help them express their true needs for a deep romantic and sexual relationship. In other words, professionals can help single men create their own private scripts that allow them to express and fulfill their unique needs for emotional fulfillment and a sense of meaningfulness in their life.

The results of our study highlight the need to recognize both the benefits and the downsides of single status. Professionals working with single men could help them acknowledge and tolerate the ambiguity inherent in singlehood (see Jackson 2018). Creating an understanding environment to explore all the pros and cons of being single may be crucial for single men to decide whether they prefer to accept their single status or want to find a partner. If singlehood is involuntary and single men would like to change it, professionals should help them find the proper strategy that acknowledges the uncertainty associated with the process of searching for a romantic partner. They could be helped by recognizing that it is necessary to be proactive, at least to some degree, and to accept possible rejection and the lack of full control over the effects of one’s pursuits. In the case of men, professionals should also help them see how they are under pressure from the social context, which tends to force men to be proactive and in control of their sexual life.

## Conclusion

The current investigation demonstrates the complicated and multifaceted nature of the experience of being single among men, and shows that singlehood can be an essential aspect of their lives. Our study reveals that single men attempt to find their own individual way of experiencing and dealing with singlehood, navigating in the context of traditional masculinity norms and gender stereotypes. In general, our findings contradict stereotypical beliefs about single men and the notion that it is much easier for women than for men to be satisfied with living single. They also indicate that single men face several challenges in sustaining emotional and developmental growth in a couple-oriented social context.

## Data Availability

The dataset generated and analyzed in the current investigation is not publicly available to honor the individual privacy of the participants, but is partially available from the corresponding author on reasonable request. The supplementary materials are available at the Open Science Repository at: https://osf.io/4ub8t/?view_only=4c44774a6e4a4a28a205f8cdfaf3bf60.
